# *Tol2* transposon-mediated transgenesis in the Midas cichlid (*Amphilophus citrinellus*) — towards understanding gene function and regulatory evolution in an ecological model system for rapid phenotypic diversification

**DOI:** 10.1186/s12861-017-0157-x

**Published:** 2017-11-23

**Authors:** Claudius F. Kratochwil, Maggie M. Sefton, Yipeng Liang, Axel Meyer

**Affiliations:** 1 Chair in Zoology and Evolutionary Biology, Department of Biology, University of Konstanz, Constance, Germany; 2 Zukunftskolleg, University of Konstanz, Constance, Germany; 3 International Max Planck Research School for Organismal Biology (IMPRS), Max Planck Institute for Ornithology, Radolfzell, Germany

**Keywords:** Transgenesis, Gene function, Transcriptional regulation, Cichlidae, *Amphilophus citrinellus*, Adaptive radiation

## Abstract

**Background:**

The Midas cichlid species complex (*Amphilophus spp.*) is widely known among evolutionary biologists as a model system for sympatric speciation and adaptive phenotypic divergence within extremely short periods of time (a few hundred generations). The repeated parallel evolution of adaptive phenotypes in this radiation, combined with their near genetic identity, makes them an excellent model for studying phenotypic diversification. While many ecological and evolutionary studies have been performed on Midas cichlids, the molecular basis of specific phenotypes, particularly adaptations, and their underlying coding and cis-regulatory changes have not yet been studied thoroughly.

**Results:**

For the first time in any New World cichlid, we use *Tol2* transposon-mediated transgenesis in the Midas cichlid (*Amphilophus citrinellus*). By adapting existing microinjection protocols, we established an effective protocol for transgenesis in Midas cichlids. Embryos were injected with a *Tol2* plasmid construct that drives enhanced green fluorescent protein (*eGFP*) expression under the control of the *ubiquitin* promoter. The transgene was successfully integrated into the germline, driving strong ubiquitous expression of e*GFP* in the first transgenic Midas cichlid line. Additionally, we show transient expression of two further transgenic constructs, *ubiquitin::tdTomato* and *mitfa::eGFP.* Transgenesis in Midas cichlids will facilitate further investigation of the genetic basis of species-specific traits, many of which are adaptations.

**Conclusion:**

Transgenesis is a versatile tool not only for studying regulatory elements such as promoters and enhancers, but also for testing gene function through overexpression of allelic gene variants. As such, it is an important first step in establishing the Midas cichlid as a powerful model for studying adaptive coding and non-coding changes in an ecological and evolutionary context.

## Background

Cichlid fishes are a textbook example for phenotypic diversity and rapid rates of speciation [1]. They are one of the most diverse groups of vertebrates with over 2000 described species [2]. Biologists have long been fascinated by these teleosts and numerous studies have been conducted on aspects of cichlid biology such as their strikingly diverse color patterns [3, 4], morphologies [5, 6] and behaviors [7, 8]. Newly-available genomic resources in combination with Quantitative Trait Loci (QTL) and molecular studies allow cichlid scientists to study the exact genetic mechanistic underpinnings of ecologically relevant traits [9, 10]. Hence, techniques from ‘model teleosts’, such as medaka (*Oryzias latipes*) and zebrafish (*Danio rerio*) [11] have to be adapted to functionally validate and analyze genotype-phenotype relationships in these new species. Molecular tools, in particular transgenesis, are effective for testing gene function and activity of *cis*-regulatory elements. In recent years, transgenesis technology has increasingly been applied to non-model organisms, especially driven by the use of the *Tol2* transposon-mediated insertion technology that strongly increases the insertion efficiency of recombinant DNA [11]. This expands this powerful toolset to organisms of evolutionary and ecological interest including sticklebacks [12], African cichlids [13] and killifish [14]. Here, we have successfully adapted and optimized *Tol2*-mediated transgenesis for the first time in a cichlid from the New World, the Midas cichlid species complex, *Amphilophus spp*.

**Fig. 1:**
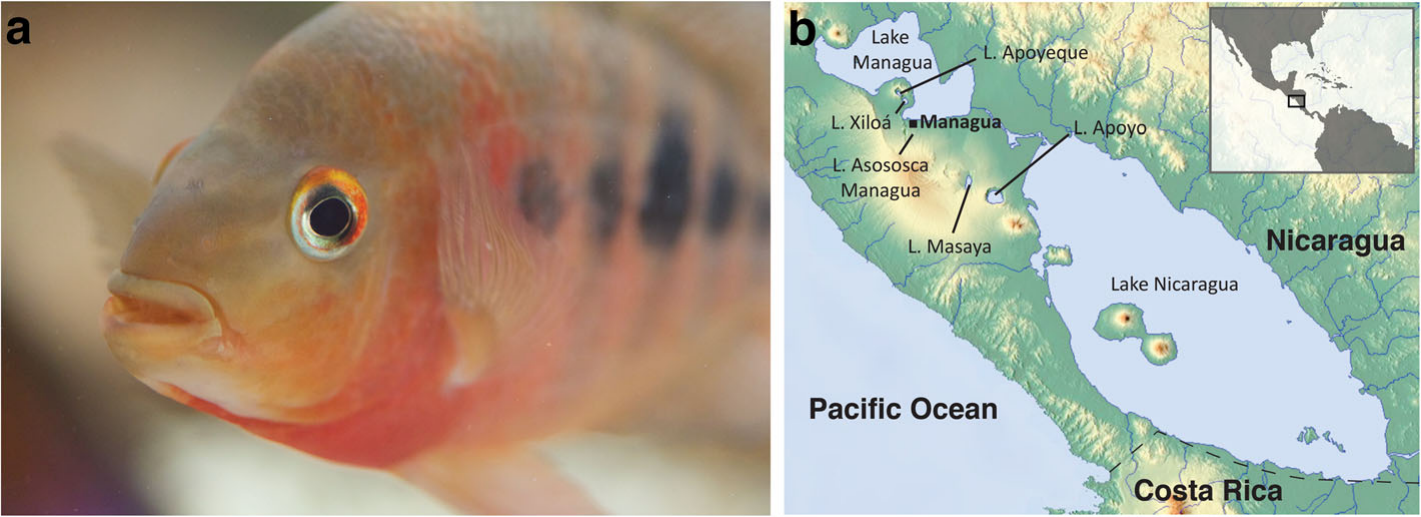
The study system. **a**-**b** Within the last 25,000 years Midas cichlids (*Amphiliphus spp.;* here *Amphilophus amarillo* from Lake Xiloá (**a**)) from Nicaragua (**b**) colonized several small crater lakes from the large lakes L. Nicaragua and L. Managua. Within the crater lakes, Midas cichlids underwent rapid and parallel adaptive evolution and formed several new species

One of the central aims of evolutionary biology is to understand how genetic changes contribute and translate to adaptive phenotypic changes. The Nicaraguan Midas cichlids (*Amphilophus spp.,* Fig. 1a) are an excellent model system for studying phenotypic diversification and how this might ultimately result in the formation of novel, distinct species. In Nicaragua, several isolated crater lakes have been colonized from the two great lakes, Lake Managua and Lake Nicaragua (Fig. 1b and [15]). The age of these crater lakes spans from 25,000 years (Lake Apoyo), to less than 1000 years (Lake Asososca Managua). These smaller crater lakes have been repeatedly colonized by Midas cichlids [16, 17]. Colonization events not only triggered diversification and adaptation to the specific Crater Lake environment [18, 19], but also gave rise to several novel species that formed both in allopatry and sympatry [5]. Each lake can be seen as a small adaptive radiation, within which species and individuals show a wide variety of morphological characteristics [20]. Several traits have been found to differ between source and crater lakes, as well as between the newly-formed species within the crater lakes [21]. These include, but are not limited to, variation in body size and shape (i.e. limnetic and benthic ecomorphs) [5, 22–24], pharyngeal jaws [5], hypertrophied lips [25], coloration [26], and visual sensitivity [19]. Midas cichlids present an excellent opportunity to determine the genetic architecture of these traits using genome scans and QTL mapping studies [9, 24]. However, bridging the gap between genotype and phenotype, and understanding how genetic changes translate to phenotypic variation, critically depends on complementary functional approaches [9]. Here, tools such as transgenesis are necessary to facilitate the discovery of the exact genetic changes and mechanisms that underlie phenotypic diversification.

Transgenesis is defined as the process of introducing new genetic information into a living organism. The development of recombinant DNA technology in the early 1970s [27] paved the way for transgenesis to become a widely-used technique in experimental biology. The first transgenic zebrafish was produced in 1988 [28]. Since then, more efficient methods of producing transgenic zebrafish have been developed using transposon-mediated insertion. The now-common *Tol2* transposable element was originally isolated from medaka, and *Tol2* transposon-mediated transgenesis [11], our method of choice, represented a significant improvement in the efficacy of transgenesis compared to previous approaches. Although the use of transgenesis in zebrafish and medaka is widespread, its use in other teleosts has been fairly limited until recently. Within the last several years, transgenesis has been successfully used in non-model organisms such as the Nile Tilapia (*Oreochromis niloticus*) [29], the haplochromine cichlid *Astatotilapia burtoni* [13], the African turquoise killifish (*Nothobranchius furzeri*) [14] and the three-spined stickleback (*Gasterosteus aculeatus*) [12]. Our study adds the Midas cichlid to this growing list of non-model teleost species.

**Fig. 2:**
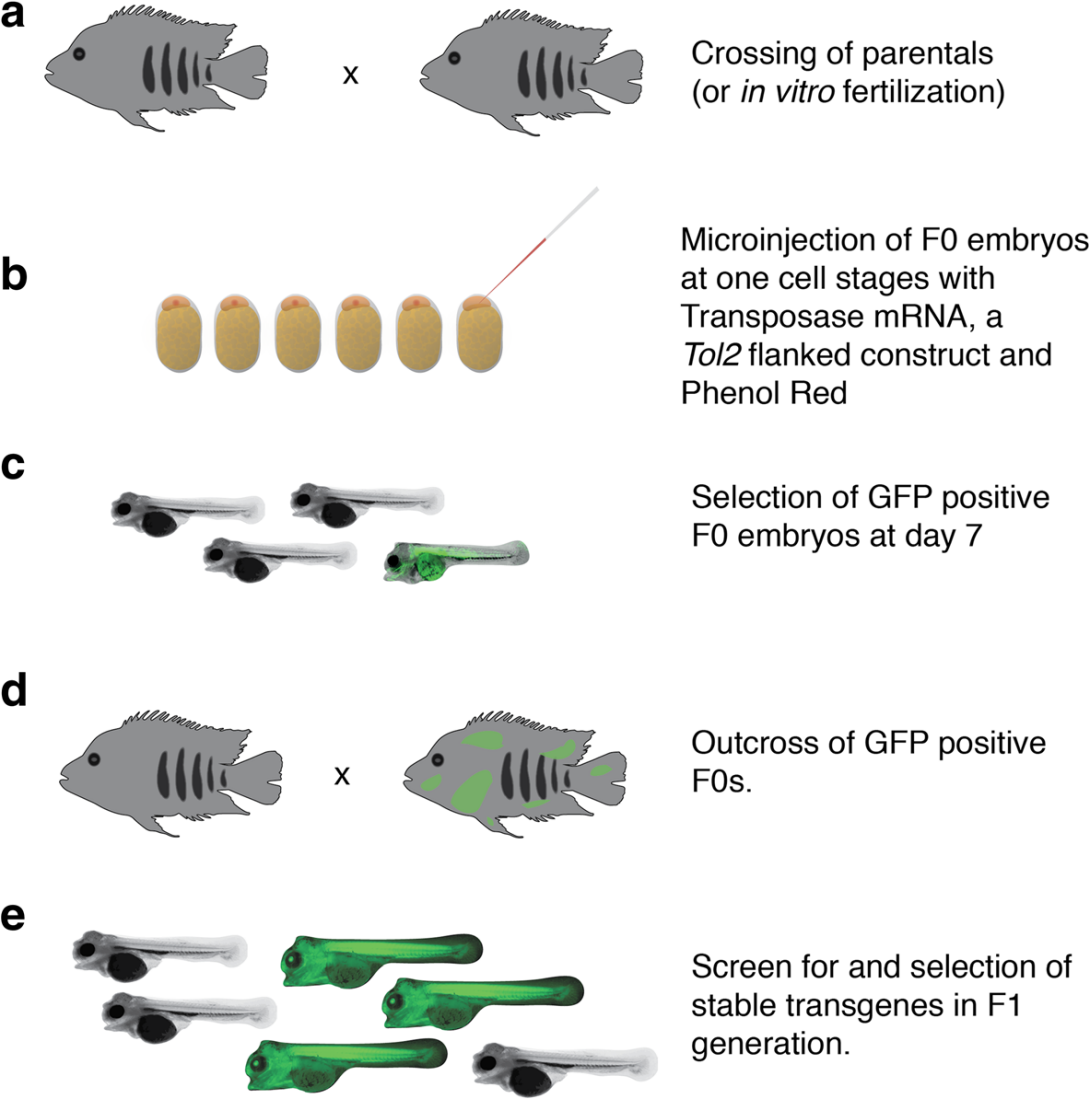
Experimental overview. **a** Midas cichlids are crossed. After successful fertilization, eggs are immediately collected. Alternatively, eggs can be fertilized in vitro. **b** Embryos at the one-cell stage are injected with a mix of Transposase mRNA, phenol red and a *Tol2* flanked DNA-construct. **c** Positive embryos show a mosaic pattern of GFP fluorescence. They are screened and selected seven days after fertilization. GFP positive larvae are raised. **d**-**e** GFP positive individuals are crossed after 9–12 months (**d**) to obtain stable transgenic Midas cichlid lines (**e**)

In this study, we show that the *Tol2* system of transgenesis can be successfully applied to the Midas cichlid (Fig. 2). We established a stable line of Midas cichlids carrying a ubiquitously expressed enhanced Green Fluorescent Protein (*eGFP*) construct (*ubi::eGFP*). For this study, we used a construct that combines the *ubiquitin (ubi)* promoter region, expressed in all eukaryotic cells, and the gene coding for *eGFP*. This construct was chosen for testing because the fluorescent reporter can be expressed in all cell types, facilitating the quantification of the presence and intensity of transgene expression in treated embryos. The transgene was successfully integrated into the germline, confirming that transgenesis, an important and versatile tool, can be used in Midas cichlids. To further demonstrate the wide applicability of this technology in Midas cichlids, we provide transient expression data for two additional constructs: 1) *ubi::tdtomato*, a construct with the red fluorescent protein tdTomato [30] under the control of the same *ubiquitin* promoter and 2) *mitfa::eGFP* that drives pigment-cell specific GFP expression under the control of the promoter of the melanoblast/melanophore marker *microphthalmia-associated transcription factor* (*mitfa*) [31, 32].

## Methods

### Fish husbandry and egg collection

**Fig. 3:**
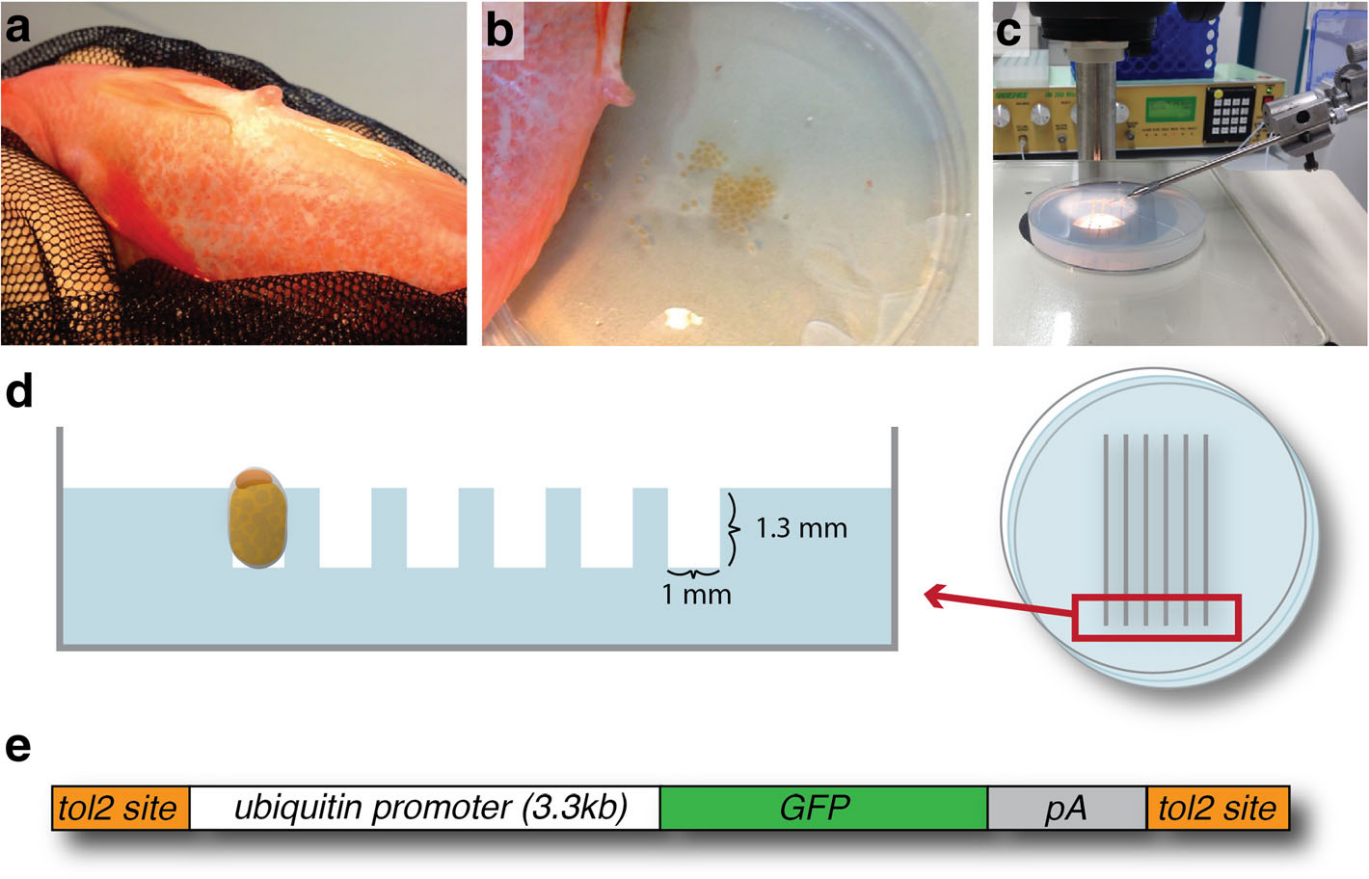
Egg stripping and microinjection. **a** Female Midas cichlid (here a golden morph of *Amphilophus xiloaensis*) with enlarged genital pore. **b** For in vitro fertilization, eggs are stripped from female fish into petri dishes. To fertilize the eggs, one or more males are stripped. Alternatively, eggs can be taken immediately after ‘natural fertilization). **c** The microinjection setup that is used for injecting the Midas cichlid embryos. **d** Orientation of eggs in custom-molded agarose injection plates. The eggs must be oriented in an upright position to allow injection precisely into or just below the cell. **e** Scheme of the construct used for the generation of the *ubi::eGFP* line

Adult Midas cichlids *(Amphilophus citrinellus)* were maintained in aquarium facilities at the University of Konstanz under constant conditions (28 ± 1 °C, 12 h dark/light cycle, pH 7.5 ± 0.5) as previously described [33]. Gravid females with fully-developed eggs ready for fertilization are identifiable by their characteristic swollen and enlarged genital pore (Fig. 3a). Eggs were stripped and fertilized (Fig. 3a, b) or taken promptly after natural fertilization, as previously described [33].

### Cloning

Transgenes were generated using the construct *pT2A_ubiquitin-eGFP-pA_pA2* (Fig. 3e). Using site-specific recombination-based cloning (multisite Gateway technology), we combined the promoter region of ubiquitin (*p5E_ubi*, Addgene ID 27320; [34]) with the Tol2-Kit constructs *383_pME-EGFP*, *302_p3E-polyA* and *394_pDestTol2pA2* [35] as well as pME-tdTomato [30]. To generate the *p5e-mitfa* vector, a 1.1kB fragment including 53 bp of 5’UTR and 1054 bp upstream of the 5’UTR were amplified from *A. citrinellus* genomic DNA using the primer pair 5′ – gat cgc tcg agC ATC TTT GTT CCT TAT CC and 5′ – gat cga cta gtT CCC TTT ATC TTG TTA GC (hybridization sequence in uppercase, leader sequence and restriction site in lowercase). The fragment was cloned into the multiple cloning site of p5e-MCS using the restriction enzymes XhoI and SpeI. *pT2A_mitfa-eGFP-pA_pA2* and *pT2A_ubiquitin-tdTomato-pA_pA2* were generated using site-specific recombination-based cloning as previously described [35].

### Microinjection

After fertilization, eggs were transferred into 2% agarose plates molded with custom-designed injection trenches (Fig. 3c, d). Using forceps, eggs were inserted into the trenches, oriented in an upright position with the animal pole on top. Injections were performed using glass capillaries (Hilgenberg, length 100 mm, outside diameter: 1.0 mm; inside diameter 0.58 mm) pulled on a Sutter P-97 Flaming/Brown Micropipette Puller. A solution composed of the plasmid construct (12.5 ng/μl), transposase (12.5 ng/μl), RNAse-free water and phenol red (1%) for visualization was co-injected into the embryos. An air pressure-driven microinjector (Narishige IM-300) was used for injections. Injection volume was adjusted to fill approximately 5% of the egg volume. The solution was injected directly into the developing one-cell stage embryo to maximize successful incorporation into the genome. Because early embryonic development in Midas cichlids proceeds relatively slowly compared to other teleost species [33], it is possible to inject 500–1000 eggs before the first cell division takes place, 90 to 100 min after fertilization.

### Maintenance of larvae, image acquisition and establishment of stable transgenic lines

After injection, eggs were transferred to new plates, with roughly 50 embryos per dish to avoid overcrowding, with fresh autoclaved water from the aquarium facility, and kept in a 28 °C incubator (HIR10M Grant, Boekel) without agitation or aeration. Embryos and larvae were previously tested in conditions with and without agitation or aeration, and these two procedures were found to have no effect on survival [33]. Every 24 h, surviving embryos were transferred to a new petri dish with clean, autoclaved tank water. At seven days post-fertilization, larvae were selected to be raised to maturity. Here, only the larvae showing strong eGFP fluorescence were kept and raised.

To prepare the embryos and larvae for photography, fish were first anesthetized with 0.04% tricaine (MS-222). They were then positioned on a slide using 3% methycellulose. Color photographs were taken with a stereomicroscope (Leica MZ10 F with Leica DMC2900 Camera) using the Leica Application Suite software 4.5.0. To improve the depth of field, we used the “Multifocus Montage” module/plugin of the Leica Application Suite software as previously described [33]. Fluorescent images were taken using the same microscope and software, with a Leica Camera (DFC3000G) and a GFP filter.

After screening for fluorescence, F0 larvae displaying widespread expression of the *ubiquitin-eGFP* transgene were raised to maturity under standard aquarium conditions. We raised ~40 F0 individuals displaying strong fluorescence, of which ten survived to adulthood. After reaching sexual maturity, Passive Integrated Transponder (PIT) tags were implanted inter-muscularly into the dorsal side of the body. Tagged males were then stripped to fertilize wild-type eggs in vitro. The fertilized eggs, referred to as the F1 generation, were screened for survival and fluorescence as described above. Of the five breeding pairs analyzed, two produced clutches with fluorescent offspring.

### Sectioning and microscopy

Larval and juvenile fish were sectioned and photographed under a fluorescence microscope. Larvae and juveniles were anaesthetized in tricaine methanosulfonate (MS-222) and fixed for two hours in 4% paraformaldehyde (PFA) in phosphate-buffered saline (PBS) at 4 °C. After fixation, the specimens were rinsed with PBS and transferred into 30% sucrose in PBS at 4 °C until the specimens sank. The samples were then embedded at 37 °C in pre-heated 11.5% gelatin / 30% sucrose in PBS for 30 min and allowed to harden at room temperature. Gel blocks were trimmed to leave ~5 mm gel on each side of the sample, then slowly lowered into 2-Methylbutane chilled by dry ice until the block froze through, and kept at −80 °C. Sections were cut at 20 μm using a cryostat microtome (HM 500 OM, Microm) at −20 °C and mounted on Superfrost™ Plus Microscope Slides (Menzel-Gläser) at room temperature. The slides were air-dried at room temperature for 30 min then rinsed three times with PBS for ten-minute intervals. The sections were counterstained with 2 μg/ml 4′,6-Diamidine-2′-phenylindole dihydrochloride (DAPI, Sigma) in PBS in dark conditions at room temperature for 20 min and rinsed three times with PBS for ten-minute periods. Slides were mounted in Mowiol mounting medium.

## Results

### Microinjection and screening process

One of the most common techniques to manipulate the genome of teleosts is through transgenesis, the integration of foreign DNA-constructs into the genome [9]. To generate stable transgenic lines, recombinant DNA has to be integrated into the germline (germline transgenesis). In teleosts, transgenesis of somatic and germ cells can be obtained most effectively by the microinjection of recombinant DNA into one-cell stage embryos. The integration can be significantly increased by co-injection of a *Tol2* insertion site-flanked DNA construct and Transposase-encoding mRNA that is readily translated and triggers DNA insertion in a cut-and-paste manner. As a first step, we sought to optimize microinjection conditions in Midas cichlids using a construct expressing a fluorescent reporter. The construct selected for use in this study was comprised of the zebrafish *ubiquitin* promoter region [34] and the *eGFP* reporter gene flanked by *Tol2* insertion sites (Fig. 3e). In zebrafish, the *ubiquitin* promoter drives strong and ubiquitous expression during all developmental stages and in all organs. Hence, it is ideal for assessing the applicability and efficacy of transgenesis.

**Fig. 4:**
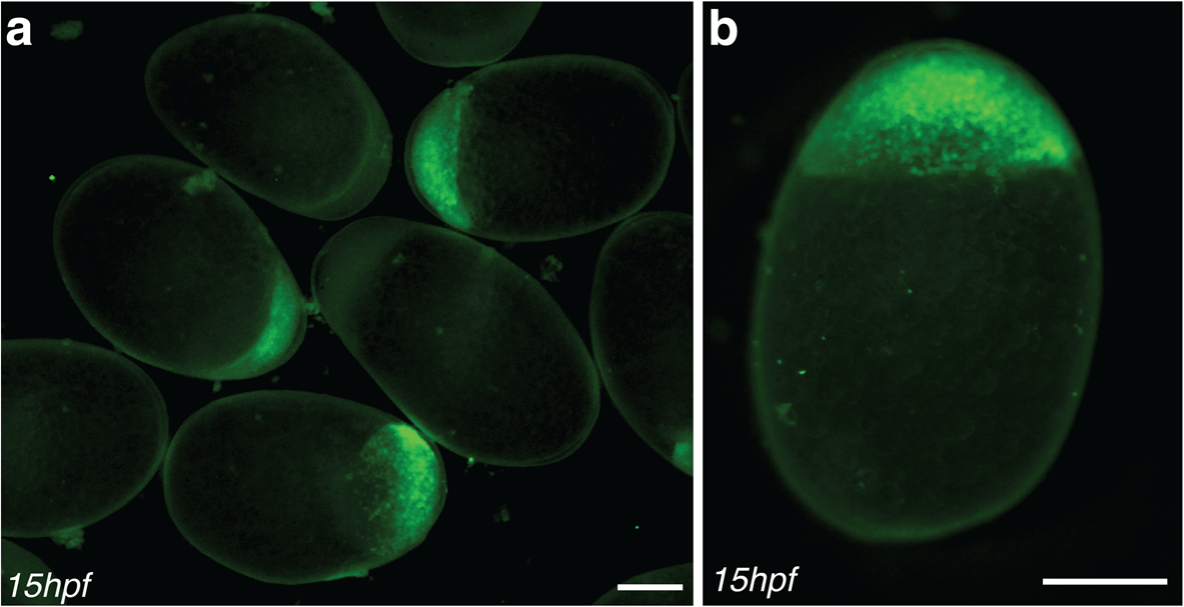
Onset of GFP fluorescence. **a-b** 15 h after fertilization (dome stage) GFP fluorescence can be readily seen and used for selecting positive embryos. Scale bars = 500 μm

**Fig. 5:**
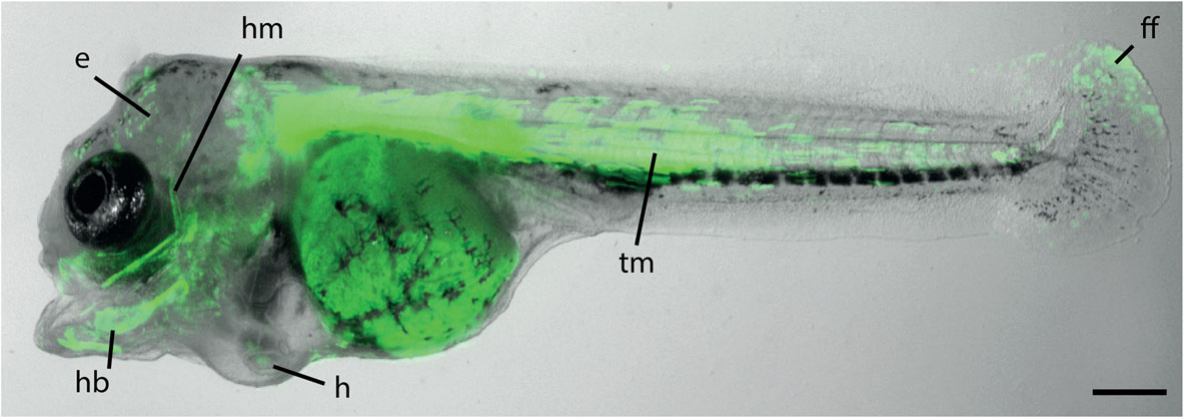
Transient expression of *ubi::eGFP*. At seven days after fertilization, fluorescence can be seen in a mosaic pattern across all tissues including trunk musculature (tm), head bones (hb) and muscles (hm), fin folds (ff), epidermis (e) and heart (h). Images are composites of brightfield and GFP-filter photographs. Scale bar = 500 μm

In contrast to the small, round eggs of zebrafish, Midas cichlids eggs are almost two times larger and have an ovoid shape that complicates precise injections. In an effort to optimize injection conditions, we produced agarose trays allowing for the alignment and fixation of embryos in an upright position with the animal pole on the top (Fig. 3d). Microinjection of a mixture of transposase mRNA, DNA, RNAse free water and Phenol red was performed directly into the cell or in the yolk slightly underneath the cell. Injections were carried out until the first cell division occurred, approx. 100 min after fertilization. Strong transient fluorescence can be readily seen at 15 h after fertilization (Fig. 4a, b). This stage corresponds to the dome stage in zebrafish at around 4 h post fertilization [33, 36]. At 7 days post fertilization (dpf), strong fluorescence can be observed in several cell types and tissues, particularly mesodermal and epidermal derivatives (Fig. 5).

### Generation and analysis of a stable *ubi::eGFP* transgenic Midas line

**Fig. 6:**
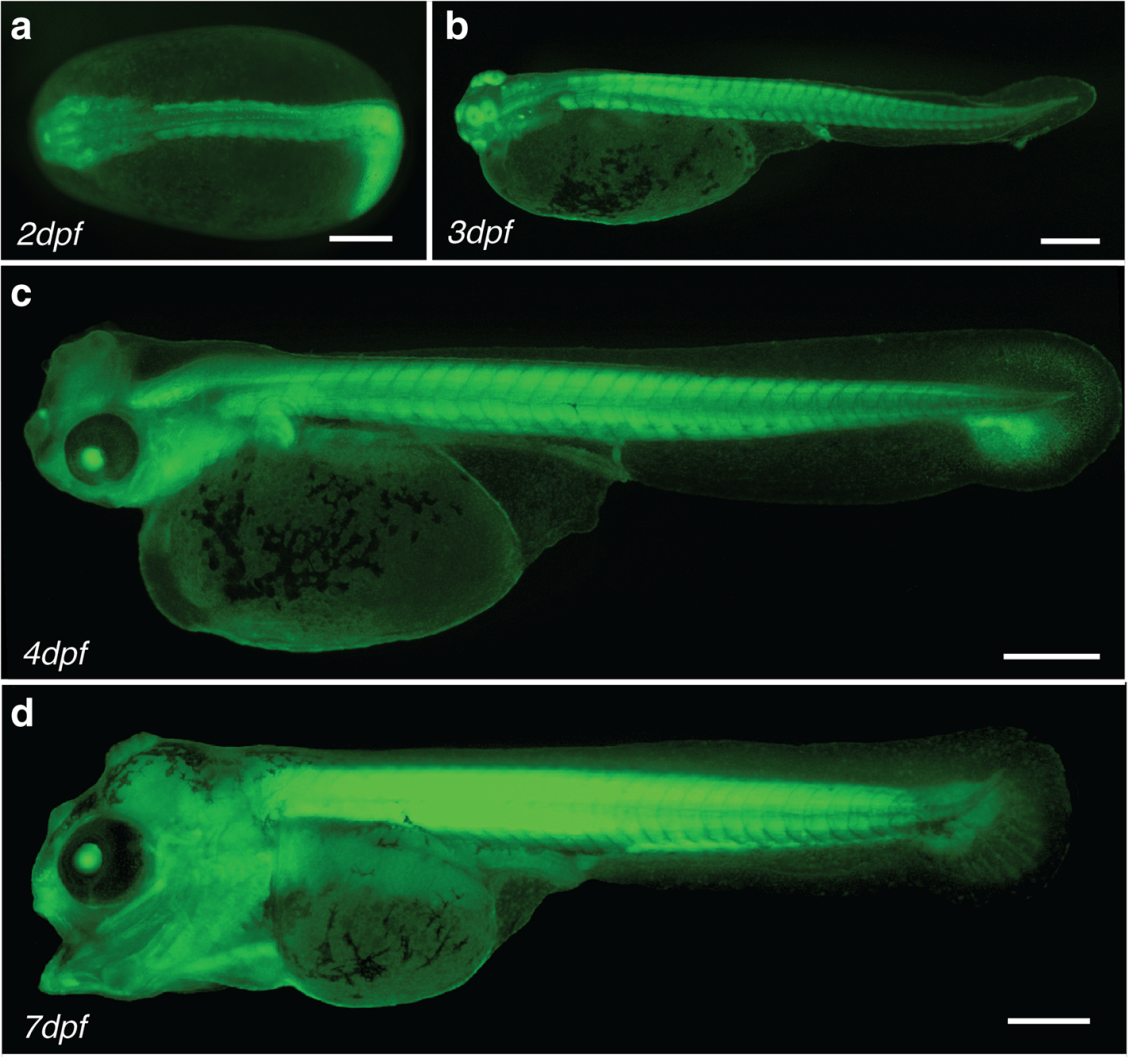
*Ubi::eGFP* F1 larvae throughout early development. **a**-**d** F1 individuals carrying the *ubi::eGFP* transgene at 2dpf (**a**), 3dpf (**b**), 4dpf (**c**) and 7dpf (**d**). Scale bars = 500 μm

**Fig. 7:**
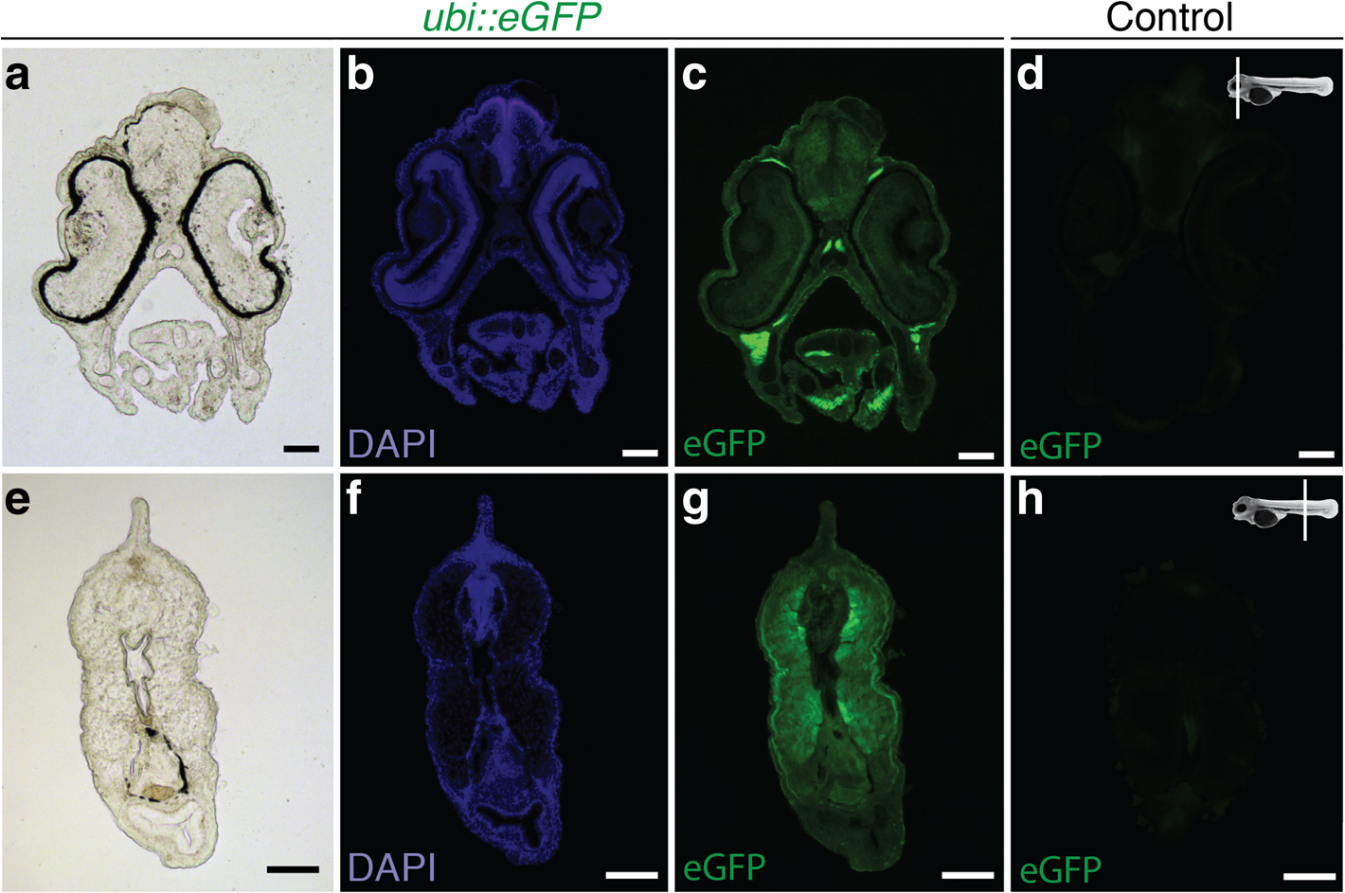
Transverse sections of *ubi::eGFP* and wild type larvae at 7dpf. **a**-**h** All larvae were stained with DAPI (**b**, **f**) and photographed under the same conditions. While F1 *ubi::eGFP* larvae (**a**, **e**) show bright fluorescence under GFP filtered light (**c**, **g**), wild types show minimal autofluorescence (**d**, **h**). Sketches indicate the location of the sections. Scale bars = 100 μm

**Fig. 8:**
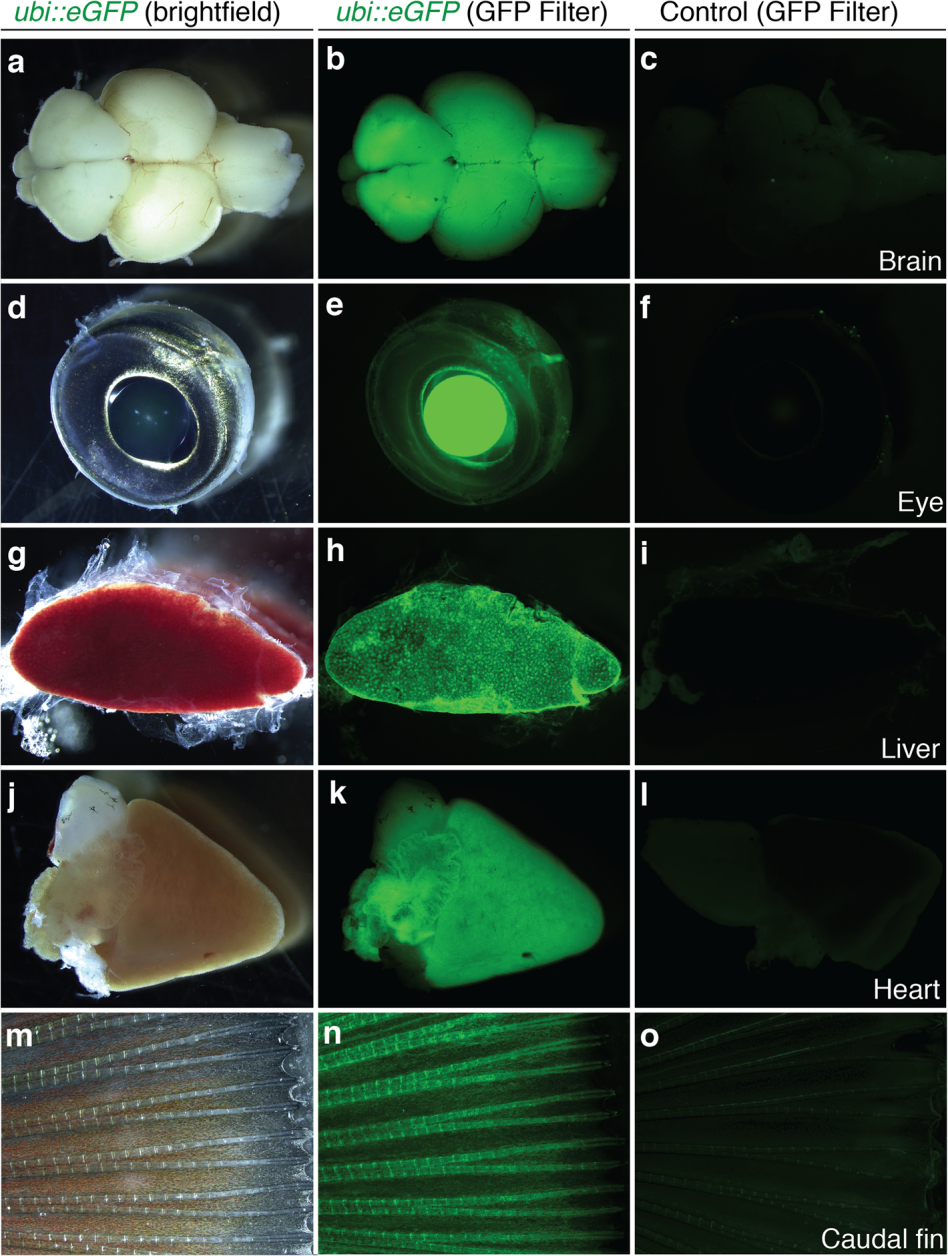
*Ubi::eGFP* transgene expression in F1 organs. **a**-**o** F1 individual shows bright fluorescence throughout the body including brain (**a**, **b**), eye (**d**, **e**), liver (**g**, **h**), heart (**j**, **k**) and fins (**m**, **n**) when viewed under fluorescent light with a GFP filter (**b**, **e**, **h**, **k**, **n**). Organs of non-transgenic fish show minimal levels of autofluorescence in every organ examined (**c**, **f**, **i**, **l**, **o**)

Fluorescent individuals were selected and raised in aquaria. Eggs of five independent mating pairs were obtained after one year and screened for fluorescence. Out of five pairs, two produced clutches with embryos ubiquitously expressing *eGFP*. Around half of the F1 generations fathered by these males were positive for eGFP fluorescence, indicating that the parental males are hemizygotic carriers of the transgenic allele. We documented eGFP fluorescence during the first seven days of development (Fig. 6). The *eGFP* expression pattern was ubiquitous, with particularly strong expression in somites (Fig. 6a, b). The expression pattern resembled that seen in the transiently expressing embryos. Next, we sectioned 7dpf embryos to show the distribution of eGFP. Notably, sections revealed that the eGFP signal is ubiquitous but not homogenous, with some tissues showing a stronger signal than others. In particular, the trunk and head muscles show a strong eGFP signal both in whole embryos (Fig. 6) and in sections (Fig. 7). In adult fish, a strong eGFP signal can be detected in all analyzed organs including brain, eye, liver, heart and fin tissue (Fig. 8). Overall, eGFP fluorescence was strong across all developmental stages and analyzed tissues.

### Transient expression patterns of two additional transgenic constructs: *ubiquitin::tdTomato* and *mitfa::eGFP*

**Fig. 9:**
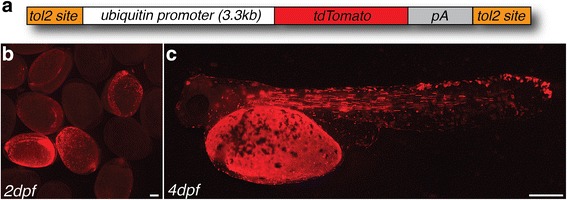
Transient expression of *ubi::tdTomato*. (**a**-**c**) Similar to *ubi::eGFP*, embryos injected with *ubi::tdTomato* (**a**) show bright fluorescence in a mosaic pattern across all tissues (2dpf, **b**; 4dpf, **c**). Scale bar = 500 μm

**Fig. 10:**
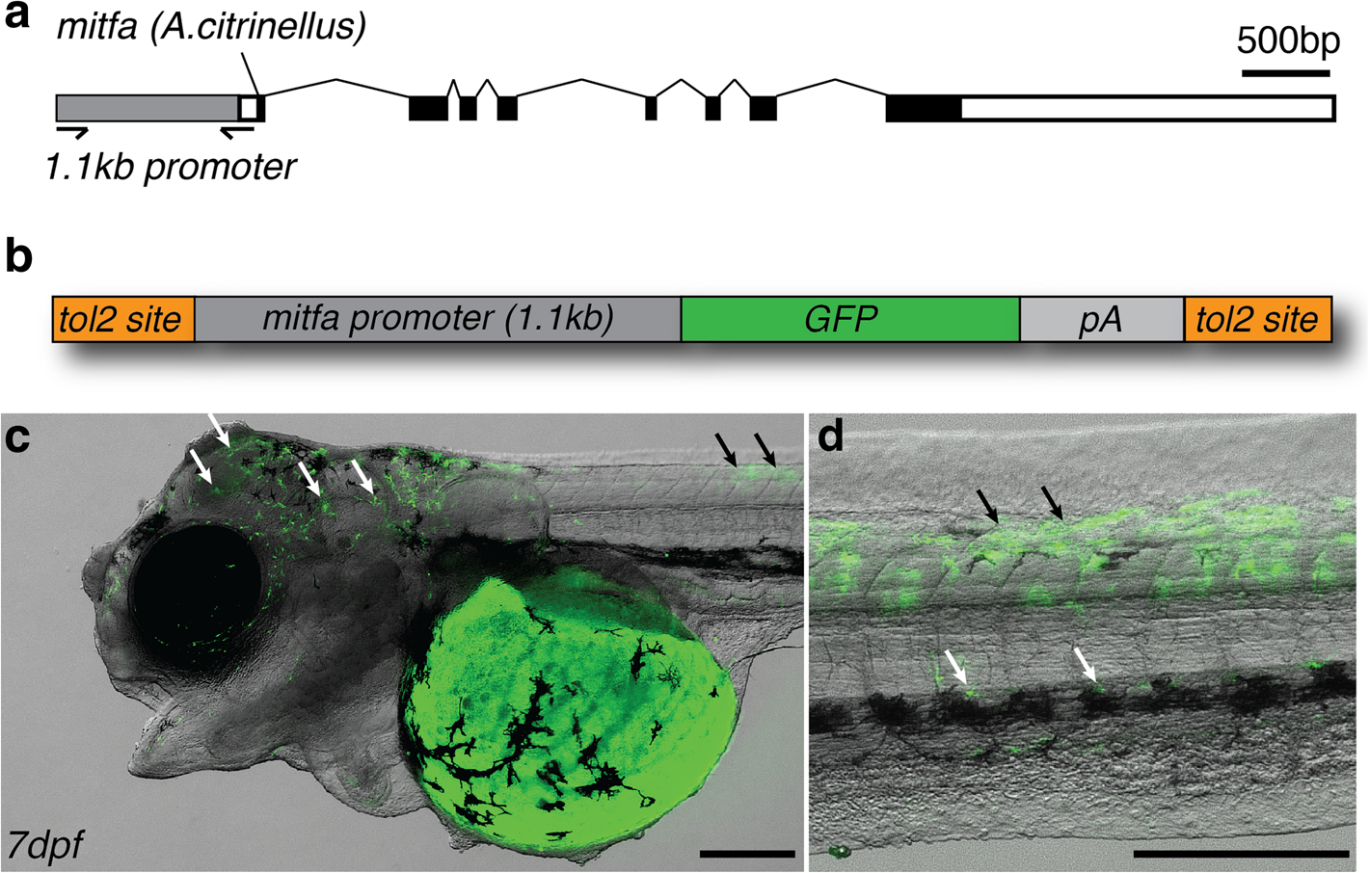
Transient expression of *mitfa::eGFP*. **a**-**b** To test a cell-type specific promoter, we cloned a 1.1kB promoter fragment of *A. citrinellus mitfa* (**a**), a melanoblast marker, upstream of eGFP (**b**). **c** At 7dpf, GFP was expressed in dendritic cells on the head (white arrows) and trunk region (black arrows). **d** On the trunk, cells expressing GFP were mainly located dorsally (black arrows), similar to expression patterns seen in zebrafish [33]. A few cells could be found more ventrally, entangled with melanophores in the ventral melanophore stripe (white arrows). Scale bar = 500 μm

To demonstrate that the transgenesis approach is widely applicable in Midas cichlids, we generated two additional constructs: *ubiquitin::tdTomato*, which uses a different (red fluorescent) reporter, and *mitfa::eGFP*, that labels pigment cells under the control of a 1.1 kb promoter element of the *microphthalmia-associated transcription factor* (*mitfa*). For *ubiquitin::tdTomato* (Fig. 9a), strong transient fluorescence is displayed in the embryos, with an expression pattern resembling that of the *ubiquitin::eGFP* construct (Fig. 9b-c). To test a more cell-specific promoter, we used the promoter sequence 1.1kB upstream of the *A. citrinellus mitfa* coding sequence (Fig. 10a) to create *mitfa::eGFP* (Fig. 10b). A similar construct using the proximal promoter sequence of zebrafish *mitfa* has previously been shown to drive melanoblast-specific expression in zebrafish embryos [31]. Indeed, GFP fluorescence could be detected in non-pigmented dendritic cells on the head and trunk (Fig. 10c-d) suggesting that the construct is able to drive expression specifically in melanoblasts (i.e. melanophore precursors).

## Discussion

In this study, we adapt existing protocols to perform transgenesis in the Midas cichlid (*Amphilophus citrinellus*). Using the *Tol2* transposon system, we produced the first transgenic Midas cichlids. As such, this work represents the first step towards testing genes and regulatory elements underlying adaptive traits in this adaptively-radiating species complex. Several important life history traits make transgenesis in this species group particularly feasible and convenient. First, unlike many of the African cichlid species, Midas cichlids are substrate-brooding fish. This facilitates the fertilization of eggs in vitro, granting more flexibility in planning experiments. Each clutch may contain over one thousand eggs, allowing for a large sample size and robust statistical analysis in any transgenic study on this species.

### Applications of transgenesis

Transgenesis enables the insertion of novel genetic information into the target genome. Therefore, it is particularly well-suited for two applications: 1) Reporter assays for testing the activity and expression pattern of cis-regulatory elements such as promoters and enhancers, and 2) overexpression experiments to analyze gene function. This methodology would therefore allow to test regulatory element candidates obtained from QTL studies or association studies [12, 37], as well as through methods such as ChIP-seq and ATAC-seq that allow for genome-wide identification of active regulatory elements [38, 39]. Gene function can also be assessed using overexpression, in which the expression of a gene of interest is increased by integrating another copy of the gene. This gene can be under the control of a ubiquitous promoter, or can be further specified in space and time using tissue-specific promoters. Overexpression can be effectively used to mimic regulatory changes that might ultimately explain phenotypic differences. On the other hand, phenotypes that result from gene loss or hypomorphic mutations affecting gene function can be rescued by the overexpression of the respective gene [9]. Cell-type specific constructs such as *mitfa::eGFP* will be especially valuable resources to improve the understanding of pigmentation phenotypes in cichlid fishes, a family known for its rich diversity of hues and color patterns.

### Advantages and pitfalls of performing transgenesis in Midas cichlids

Several factors determine the suitability of a teleost species for transgenesis studies. Critical factors are 1) frequent breeding under lab conditions, 2) the possibility of raising larvae under lab conditions, 3) possibility to obtain one to two-cell stage embryos, 4) large clutch sizes, 5) regular breeding times, 6) a penetrable chorion that permits microinjection and 7) short generation times to obtain F1 individuals. For many ecological model systems, one or more of these factors hampers efficient transgenesis. In sticklebacks, an excellent system for analyzing gene function and regulatory divergence, transgenesis is particularly complicated by seasonal breeding behavior and small clutch sizes [37]. Likewise, African cichlids are an excellent model system for understanding phenotypic diversification, but suffer from drawbacks regarding transgenesis. In the case of the African cichlids, the combination of small clutch sizes, mouth-brooding and the difficulty of timing fertilization make the application of transgenesis at large scales prohibitively challenging. Midas cichlids exhibit several traits that make transgenesis a suitable tool for this model system. A few days before fertilization, Midas cichlids form monogamous pairs [40]. At the time the genital papillae swells, fertilization can be predicted to occur within the next 24 h. Consequently, eggs can be collected directly after natural fertilization, or artificially fertilized as previously described [33]. The clutches are large (up to 1500 or more eggs) and develop relatively slowly. Larvae are robust and can be easily raised in tap water under lab conditions [33]. One of the major drawbacks of Midas cichlids is their long generation time, which can range from nine to twelve months. While the aforementioned advantages ease transient analysis, long generation times make it time- and space consuming to obtain stable transgenes.

### From Midas genotypes to Midas phenotypes

Midas cichlids are an excellent example of rapid phenotypic changes. This includes adaptive variation in body shapes (i.e. limnetic and benthic forms) [5, 24], hypertrophied lips [25], teeth and pharyngeal jaws [5], the gold/dark polymorphism of Midas cichlids [26], and visual sensitivity [19]. Increasing genomic and transcriptomic resources facilitate the discovery of more and more genotype-phenotype relationships. However, to further understand which genetic elements contribute to phenotypic variation, it is essential to pinpoint and validate their functional relevance. Testing of regulatory elements using GFP transgenesis assays and overexpression of target genes [41] via transgenesis are important tools that will bring researchers closer to understanding the relationship between genotype and phenotype.

## Conclusion

Transgenesis is a key technology for understanding the genetic and molecular basis of adaptive traits. For the first time, we used *Tol2*-mediated transgenesis in the Midas cichlid, a model system for fast and repeated parallel evolution of adaptive phenotypes. This technological advancement opens up new possibilities for studying the genotypic and molecular basis of adaptive traits in Midas cichlids, and provides a workflow for other substrate brooding cichlids and teleosts. We anticipate that the use of transgenesis in Midas cichlid will contribute novel insights into the genetic underpinnings of early stages of diversification.

## Abbreviations

dpf: days post fertilization
*eGFP*: enhanced green fluorescent protein
*ubi*: ubiquitin
